# Evaluating the Indirect Costs of Care Associated with Salvage Chemotherapy for Relapsed and Refractory Aggressive-Histology Lymphoma: A Subset Analysis of the Canadian Cancer Trials Group (CCTG) LY.12 Clinical Trial

**DOI:** 10.3390/curroncol28020119

**Published:** 2021-03-17

**Authors:** Anca Prica, Annette E. Hay, Michael Crump, Nicole Mittmann, Lois E. Shepherd, Ralph M. Meyer, Kevin I. Imrie, Nancy Risebrough, Marina Djurfeldt, Bingshu E. Chen, Matthew C. Cheung

**Affiliations:** Canadian Cancer Trials Group, Queen’s University, Kingston, ON K7L 3N6, Canada; ahay@ctg.queensu.ca (A.E.H.); Michael.Crump@uhn.ca (M.C.); Nicole.Mittmann@cadth.ca (N.M.); lshepherd@ctg.queensu.ca (L.E.S.); meyerr@hhsc.ca (R.M.M.); Kevin.Imrie@sunnybrook.ca (K.I.I.); nancy.risebrough@gmail.com (N.R.); MDjurfeldt@ctg.queensu.ca (M.D.); bechen@ctg.queensu.ca (B.E.C.); Matthew.Cheung@sunnybrook.ca (M.C.C.)

**Keywords:** lymphoma, autologous stem cell transplantation, cost-effectiveness analysis

## Abstract

We conducted an analysis of indirect costs alongside the LY.12 randomized trial in patients with relapsed or refractory (R/R) aggressive non-Hodgkin lymphoma (NHL). Lost productivity data for Canadian patients and caregivers in the trial were collected at baseline and with each chemotherapy cycle pre-transplant, using an adapted Lost Productivity questionnaire. Mean per patient indirect costs were CAD 2999 for patients in the GDP arm and CAD 3400 in the DHAP arm. A substantial majority was not working or had to reduce their workload during this treatment time. Salvage chemotherapy for R/R aggressive NHL is associated with significant indirect costs to patients and their caregivers.

## 1. Introduction

Aggressive lymphomas are curable cancers at presentation, but 20–30% of patients will experience progression. For such patients, salvage chemotherapy followed by autologous stem cell transplantation (ASCT) remains one of the only curative options [1]. This is a disease mostly affecting older patients, however about 45% of patients are diagnosed between the ages of 20 and 64, which for most is during their productive working years. Furthermore, patients eligible for salvage therapy and ASCT are generally 70 years of age or less. Non-Hodgkin lymphomas (NHL) can thus have a significant financial impact to patients, particularly on work productivity, leading to absenteeism, presenteeism, short-term and long-term disability, as well as need for paid and unpaid caregiving costs, all translating into indirect costs.

The economic burden of NHL consists of both direct and indirect costs. Few studies have examined these costs in the lymphoma population. A recent study examined the indirect costs and lost productivity associated with NHL in commercially insured working adults in the US, using a large commercial insurance claims database, and linking this to clinical diagnoses and outcomes [2]. When comparing 168 NHL patients to 508 controls and adjusting for relevant variables, NHL patients incurred significantly more estimated mean days of workplace productivity loss (31.99 days, *p* < 0.001) and significantly more estimated mean indirect costs (CAD 6302.34; 95% CI: CAD 4973.40, CAD 7631.28; *p* < 0.001) in the 12 months post-diagnosis.

The randomized CCTG LY.12 trial demonstrated that gemcitabine, cisplatin and dexamethasone (GDP) was non-inferior with respect to response rates and survival to dexamethasone, cytarabine, cisplatin (DHAP), with or without rituximab, in patients with relapsed or refractory (R/R) aggressive non-Hodgkin lymphoma (NHL) prior to autologous stem cell transplantation (ASCT) [3]. DHAP was also associated with more toxicity and inferior quality of life compared to GDP. A cost-utility analysis based on direct medical costs also proved GDP to be associated with both lower direct costs (CAD 19,961 vs. CAD 34,425) and similar quality-adjusted outcomes, and as such, is the preferred salvage regimen in this patient population [4].

The LY.12 trial allowed for the direct evaluation of costs, including indirect costs related lost productivity and formal and informal caregiving. We hypothesized that these costs will be significant in this younger patient population and lower in the GDP arm given its outpatient administration and potentially fewer lost workdays and need for caregiving. We conducted an analysis of such costs based on the trial data.

## 2. Materials and Methods

Resource utilization data were collected for all Canadian patients in the trial as part of economic analysis that was embedded into the trial protocol. Lost productivity data for both patients and caregivers were collected at baseline and with each chemotherapy cycle pre-ASCT (up to 3 cycles), from a proportion of the Canadian patients, using an WPAI-adapted Lost Productivity questionnaire [5]. The clinical and economic evaluations both included intent-to-treat analyses. Patients were included if they completed the baseline questionnaire and at least one follow-up questionnaire. If there was missing data, values were imputed from the patient’s last completed survey instrument. The research ethics boards of all participating centers approved the trial, and all participants provided informed consent.

The human capital approach was utilized to calculate lost productivity costs using published Canadian salary standards according to field of employment [http://www.statcan.gc.ca] (accessed on 18 October 2020) [6]. We assumed that a full-time week is 40 h, part-time week is 20 h, and that a workday is 8 h. Patients who at baseline were on sick leave, disability, unemployed, retired, or homemakers were assumed based on expert consensus to contribute to society at a rate that is 50% of the 2012 Canadian general income wage.

Costs are presented in 2020 Canadian dollars (1 CAN$ = 0.79 US$) from a societal subset perspective (indirect costs inclusive of lost productivity and care costs) [7], using the Consumer Price Index (www.bankofcanada.ca) (accessed on 18 October 2020) and were not discounted, given the brief time horizon between random assignment and stem cell mobilization [4]. Costs were disaggregated to highlight the costs of patient lost productivity, paid caregiving hours and lost productivity from family members providing unpaid caregiving, and these were compared between the two treatment strategies. All statistical tests were two-sided, and comparisons of non-parametric data were completed using the Wilcoxon rank sum test.

## 3. Results

There were 619 Canadian and international patients in the trial, 374 completed at least 2 lost productivity (LP) instruments (60%) and were included in this analysis. There were no significant differences between the two arms with respect to demographics and lymphoma disease characteristics. As well, the lost productivity subset of patients was representative of the whole Canadian cohort (Table 1).

Mean per patient indirect costs over the period of 2–3 cycles of chemotherapy were CAD 2999 (95% CI CAD 2545 to CAD 3454; Median CAD 2238; IQR CAD 1201–CAD 3809) for patients in the GDP arm, an outpatient chemotherapy regimen, and CAD 3400 (95% CI CAD 2637 to CAD 4164; Median CAD 2240; IQR CAD 1185–CAD 4131) for patients in the DHAP arm, a 3-day inpatient chemotherapy regimen (Table 2). There was no statistically significant difference in mean costs detected (difference CAD 401; *p* = 0.63). At salvage chemotherapy start, 66% of all patients were non-workers (69% of patients in the GDP arm and 63% in the DHAP arm). Of those that were not working, 41% were on sick or disability leave, 7% were unemployed and 18% were retired. Eighteen percent of patients were still working full-time, 11% part-time, and 5% were homemakers. Of those still working full time at baseline, 3.5% reported decreasing their workload (part-time or stopped working) in the GDP arm, compared with 7.4% in the DHAP arm. Patients in both treatment arms reported substantial limitations in their usual activities, with approximately 50% impairment due to their health status.

The different cost components were disaggregated. Lost productivity of patients accounts for 65% of total indirect costs. Mean patient lost productivity costs were CAD 2155 (Median CAD 1729; IQR CAD 977–CAD 2916) with GDP and CAD 2013 (Median CAD 1581; IQR CAD 892–CAD 2597) with DHAP (*p* = 0.33). Mean care costs, including both paid and unpaid assistance, were CAD 855 (95% CI CAD 538 to CAD 1171; median CAD 4; IQR CAD 0–CAD 922) with GDP vs. CAD 1398 (95% CI CAD 707 to 2090; median CAD 99; IQR CAD 0–CAD 1570) with DHAP (mean difference: CAD 543; *p* = 0.07). Over the period of 2–3 cycles of salvage chemotherapy, patients in the DHAP arm paid for significantly more hours (21.3 h; median 0 h; IQR 0–16h) compared with the GDP arm (14.9 h; median 0 h; IQR 0–0.5 h), (*p* = 0.001),

Thirty-nine pts (10.4%) did not have any unpaid caregivers, while the rest were cared for by a combination of: a spouse (68.5%), child/parent (34.8%), other relative (19.5%), friend (16.3%) or neighbor (2.9%). The mean number of unpaid caregiver hours were 24.9 h with GDP (95% CI 14.2 to 35.5; median 0 h; IQR 0–24 h) compared to 42.7 h with DHAP (95% CI 15.1 to 70.3; median 0 h; IQR 0–36 h).

## 4. Discussion

The CCTG LY12 trial demonstrated that, in relapsed/refractory aggressive lymphoma patients, GDP was not only non-inferior to DHAP with respect to responses pre-ASCT, but it is associated with less toxicity, improved quality of life, and dominance when it comes to direct costs and quality-adjusted outcomes [4]. In this follow-up work, we demonstrated that salvage chemotherapy for R/R aggressive NHL is overall associated with substantial indirect costs to the patients and their caregivers, with a majority (approximately 65% of patients) not working or having to reduce their workload during this treatment time. This was likely due to disease burden and symptoms, as well as ongoing decreased workload as recovering from previous treatment, in the case of primary refractory patients, although a proportion of patients were already retired. Over the 6 to 10 week period that patients underwent salvage chemotherapy, the average indirect costs were at least CAD 3000 per patient, which appears substantially more than reported in other cancers, if adjusting for observation time. In an analysis examining the indirect costs and lost productivity associated with a new diagnosis of NHL, in commercially insured working adults in the US, the mean indirect costs in the 12 months post-diagnosis were USCAD 12,741, mostly driven by absenteeism and disability [2]. A study of breast cancer patients determined indirect costs related to absenteeism and short-term disability in the first year post-diagnosis to be approximately CAD 8000 [8,9]. These results suggest that the lost productivity impact to patients and their employers is large, as many of the patients are not working at the start of salvage chemotherapy, presuming that they were productively employed prior to their relapsed disease status. As well, this data only captures the limited time period of salvage chemotherapy, but these financial impairments would be anticipated to continue through the stem cell transplant process. As such the indirect costs to patients and society are likely much larger. This has implications for strategies to support patients and their families, and for employers in planning their workforce.

Although there were no significant differences in all indirect costs between the two treatment arms, the mean paid caregiving costs were higher with the DHAP arm. However, the distribution of hours used is of interest, as it appears that the majority of patients did not use any hours (given the median of zero hours and IQR distribution), with a smaller proportion of patients requiring a large number of paid and unpaid hours in both arms. This is likely related to large disease and symptom burden in patients not responding to salvage therapy, as well as patients suffering from severe side effects from chemotherapy. Informal caregiving, often mostly provided by family members, can lead to economic burden (i.e., providing economic support), occupational burden (i.e., missing work) and psychosocial burden (e.g., anxiety, depression, stress), and these are important indirect costs to capture and understand.

There are several limitations to this analysis. It has been suggested that trial-based interventions are more extensive and more costly than treatments in routine practice, thus indirect costs may be different if less time at the treatment centre is needed in real-life [10]. However, the LY.12 protocol minimized any incremental interventions beyond standard-of-care. The indirect costs captured were calculated from baseline through the salvage chemotherapy cycles. However, the majority of patients were not working at baseline, and we assumed these patients contribute to society at at a rate that is 50% of the Canadian general income wage, which is one indirect costs analysis approach to take. Thus, lost productivity costs may overall be underestimated. Concurrently, as a majority of patients were not working at baseline, it is likely a proportion of the lost productivity costs are due to disease burden and previous treatment effects, rather than the salvage chemotherapy itself.

## 5. Conclusions

Salvage chemotherapy for R/R aggressive NHL is associated with substantial indirect costs to the patients and their caregivers, with a large majority not working or having to reduce their workload during this treatment time. Although there were no significant differences in all indirect costs between the two treatment arms, the paid and unpaid caregiving costs did appear higher with the DHAP arm. These data potentially provide further support to GDP being the preferred treatment approach in this patient population and interventions to financially support patients and families through this time are warranted.

## Figures and Tables

**Table 1:** Lost productivity cohort of pts vs. the rest of LY.12 pts.

		Pts in Lost Productivity Cohort *n* = 374 (%)	Rest of LY.12 pts *n* = 245 (%)	TOT *n* = 619 (%)
Gender (*p* = 0.74)	Male	231(61.8)	148(60.4)	379(61.2)
Race (*p* = 0.86)	White	320(85.6)	203(82.9)	523(84.5)
	Black/African American	11(2.9)	6(2.4)	17(2.7)
	Asian	28(7.5)	(7.3)	46(7.4)
	Other	12(3.7)	5(2.0)	19(3.1)
Age (*p* = 0.81)	Mean (years)	53.04	52.68	52.9
	Std (years)	10.24	11.18	10.62
	Median (years)	55.2	54.6	54.9
Age (*p*= 0.41)	>60 years of age	102(27.3)	75(30.6)	177(28.6)
ECOG (*p* = 0.06)	0	147(39.3)	110(44.9)	257(41.5)
	1	183(48.9)	95(38.8)	278(44.9)
	2	35(9.4)	29(11.8)	64(10.3)
	3	9(2.4)	11(4.5)	20(3.2)
Disease stage on study (*p* = 0.78)	I	31(8.3)	20(8.2)	51(8.2)
	II	84(22.5)	57(23.3)	141(22.8)
	III	99(26.5)	56(22.9)	155(25.0)
	IV	160(42.8)	112(45.7)	272(43.9)
Number of extranodal sites (*p* = 0.9)	0	164(43.9)	106(43.3)	270(43.6)
	1	112(29.9)	78(31.8)	190(30.7)
	2	62(16.6)	36(14.7)	98(15.8)
	≥3	36(9.6)	25(10.2)	61(9.9)
rIPI score at baseline (*p* = 0.23)	0, 1	134(35.8)	96(39.2)	230(37.2)
	2	117(31.3)	61(24.9)	178(28.8)
	≥3	123(32.9)	88(35.9)	211(34.1)
Prior response (*p* = 0.02)	Response < 1 year	149(39.8)	112(45.7)	261(42.2)
	Response > 1 year	114(30.5)	50(20.4)	164(26.5)
	Stable/progressive disease	111(29.7)	83(33.9)	194(31.3)

**Table 2:** Indirect costs associated with the two treatment arms.

	GDP *n* = 198	DHAP *n* = 176	Mean Difference	*p*-Value
Indirect costs	CAD 2999	CAD 3400	CAD 401	*p* = 0.63
Lost productivity costs	CAD 2155	CAD 2013	CAD -142	*p* = 0.33
Care costs (paid + unpaid assistance)	CAD 855	CAD 1398	CAD 543	*p* = 0.07
Paid assistance hours	14.85	21.3	6.45	*p* = 0.001
Unpaid assistance hours	24.9	42.7	17.8	*p* = 0.44
Activity impairment	51.6%	49.4%	−2.2%	*p* = 0.38

Footnote: GDP: Gemcitabine, Dexamethasone and Cisplatin; DHAP: Dexamethasone, Cytarabine and Cisplatin; NS: no significance.

## Data Availability

The Canadian Cancer Trials Group is committed to responsible data sharing. See https://www.ctg.queensu.ca/ (accessed on 18 October 2020) or contact datasharing@ctg.queensu.ca.
